# MALT1-Dependent Cleavage of HOIL1 Modulates Canonical NF-κB Signaling and Inflammatory Responsiveness

**DOI:** 10.3389/fimmu.2021.749794

**Published:** 2021-10-14

**Authors:** Shan-Yu Fung, Henry Y. Lu, Mehul Sharma, Ashish A. Sharma, Aabida Saferali, Alicia Jia, Libin Abraham, Theo Klein, Michael R. Gold, Luigi D. Noterangelo, Christopher M. Overall, Stuart E. Turvey

**Affiliations:** ^1^ Department of Pediatrics, British Columbia Children’s Hospital and The University of British Columbia, Vancouver, BC, Canada; ^2^ Key Laboratory of Immune Microenvironment and Disease (Ministry of Education), Province and Ministry Co-Sponsored Collaborative Innovation Center for Medical Epigenetics and Department of Immunology, School of Basic Medical Sciences, Tianjin Medical University, Tianjin, China; ^3^ Experimental Medicine Program, Faculty of Medicine, The University of British Columbia, Vancouver, BC, Canada; ^4^ Department of Pathology, Case Western Reserve University, Cleveland, OH, United States; ^5^ Channing Division of Network Medicine, Brigham and Women’s Hospital, Harvard Medical School, Boston, MA, United States; ^6^ Department of Microbiology and Immunology, The University of British Columbia, Vancouver, BC, Canada; ^7^ Department of Analytical Solutions, Ducares/Triskelion BV, Utrecht, Netherlands; ^8^ Laboratory of Clinical Immunology and Microbiology, National Institute of Allergy and Infectious Diseases, National Institutes of Health, Bethesda, MD, United States; ^9^ Department of Biochemistry and Molecular Biology, Department of Oral Biological and Medical Science, Center for Blood Research, The University of British Columbia, Vancouver, BC, Canada

**Keywords:** HOIL1 cleavage, LUBAC, MALT1, NF-κB, regulation of inflammation

## Abstract

Nuclear factor kappa B (NF-κB) is a critical transcription factor involved in regulating cell activation, inflammation, and survival. The linear ubiquitin chain assembly complex (LUBAC) which consists of HOIL1, HOIP, and SHARPIN, catalyzes the linear ubiquitination of target proteins—a post-translational modification that is essential for NF-κB activation. Human germline pathogenic variants that dysregulate linear ubiquitination and NF-κB signaling are associated with immunodeficiency and/or autoinflammation including dermatitis, recurrent fevers, systemic inflammation and enteropathy. We previously identified MALT1 paracaspase as a novel negative regulator of LUBAC by proteolytic cleavage of HOIL1. To directly investigate the impact of HOIL1 cleavage activity on the inflammatory response, we employed a stable transduction system to express and directly compare non-cleavable HOIL1 with wild-type HOIL1 in primary HOIL1-deficient patient skin fibroblasts. We discovered that non-cleavable HOIL1 resulted in enhanced NF-κB signaling in response to innate stimuli. Transcriptomics revealed enrichment of inflammation and proinflammatory cytokine-related pathways after stimulation. Multiplexed cytokine assays confirmed a ‘hyperinflammatory’ phenotype in these cells. This work highlights the physiological importance of MALT1-dependent cleavage and modulation of HOIL1 on NF-κB signaling and inflammation, provides a mechanism for the autoinflammation observed in MALT1-deficient patients, and will inform the development of therapeutics that target MALT1 paracaspase and LUBAC function in treating autoinflammatory skin diseases.

## Introduction

The transcription factor nuclear factor κB (NF-κB) is a critical mediator of inflammation, immunity and malignancy (1, 2). The study of humans with pathogenic variants affecting key elements of the NF-κB signaling cascade has provided key insights into the biology of this system (3). Today we know that upon activation, NF-κB drives cellular activation, proliferation, and survival by modulating gene expression, cytokine production and signaling pathways. NF-κB can be induced by diverse innate and adaptive stimuli through the canonical (classical) or noncanonical (alternative) pathways. Major activators of the canonical pathway include signals from pathogen-associated molecular patterns (PAMPs) or damage-associated molecular patterns (DAMPs), tumor necrosis factor-α (TNF-α), interleukin-1β (IL-1β), and lymphocyte antigen receptor signaling (1). In contrast, non-canonical stimuli [*e.g.* receptor activator of nuclear factor kappa-B ligand (RANKL)] cooperate with the canonical NF-κB pathway (4). Given the central role of NF-κB, aberrant activation is associated with various pathological states, including inflammatory diseases, autoimmunity, immuno-deficiency, and cancer (2, 5, 6).

The canonical pathway of NF-κB is regulated by a variety of post-translational modifications, including phosphorylation and various topographies of ubiquitination. Classically, the NF-κB complex (consisting of a heterodimer of p65 and p50) is maintained in the cytoplasm by its inhibitor IκBα. Upstream signaling drives the activation of the IκB kinase (IKK) complex (IKKα/β/γ), which phosphorylates IκBα, triggering K48-linked polyubiquitination by SCF (Skp, Cullin, F-box containing complex)β^-TrCP^ E3 ubiquitin ligase, leading to IκBα proteasomal degradation (7). Release of NF-κB inhibition allows NF-κB translocation into the nucleus to regulate gene expression.

Non-degradative ‘regulatory’ forms of ubiquitination such as M1-linked (or linear) and K63-linked polyubiquitin have also emerged recently as critical regulators of NF-κB activation by linking signaling proteins/complexes (3, 8). In response to TNF-α or IL-1β stimulation, the linear ubiquitin chain assembly complex (LUBAC), consisting of heme-oxidized IRP2 ligase 1 (HOIL1), HOIL1 interacting protein (HOIP), and SHANK-associated RH domain interacting protein (SHARPIN), catalyzes the addition of linear ubiquitin (M1-Ub) chains onto key substrates to promote NF-κB activation (9, 10). One such substrate is IKKγ (or NEMO), the regulatory subunit of the IKK complex (11, 12). Since IKKγ possesses an UBAN (ubiquitin binding domain in ABIN and NEMO) domain that can bind M1-Ub with high affinity (13, 14), this promotes IKK complex oligomerization, autophosphorylation, and activation of IKKα/β, ultimately catalyzing IκBα phosphorylation and degradation (15). Proof of the important but intricate regulatory nature of this complex is the existence of human patients with germline pathogenic variants impairing the expression or regulation of LUBAC or M1-Ub who present with a combination of immunodeficiency and autoinflammation (16–22). For example, patients with deficiency of OTU deubiquitinase with linear linkage specificity (OTULIN), which causes a condition referred to as otulipenia, lack the ability to remove linear Ub chains causing immune dysregulation and systemic autoinflammation (21). In particular, autoinflammatory skin manifestations are significant, including neutrophilic dermatitis and panniculitis. This provides further support for the integral role of M1-Ub in regulating cell death-associated inflammation and controlling skin pathologies (23–25).

A number of mechanisms have been identified to regulate LUBAC activity. Deubiquitinases (DUBs) that specifically remove the M1-linked polyubiquitin chains (e.g., OTULIN and CYLD) reverse the LUBAC activity (26, 27). The stability of the LUBAC structure requires the interaction between each LUBAC component to form the trimolecular complex, and this is strongly associated with the protein level of HOIP, the catalytic center for linear ubiquitination (28). Deficiency in any one of the LUBAC components will affect the protein level of the other two, and hence down-regulates the LUBAC activity. This phenomenon was clearly documented in the reported HOIL1- and HOIP-deficient patients (16, 17, 19). More recently, additional regulatory roles for HOIL1 have been identified. HOIL1 conjugates monoubiquitin onto all LUBAC subunits, followed by HOIP-mediated conjugation of linear chains onto monoubiquitin, and these linear chains attenuate the functions of LUBAC (24). In addition, it has been found that HOIL1 is an atypical E3 ubiquitin ligase that forms oxyester bonds between the C-terminal carboxylate of Ub and serine and threonine residues in its substrates (29). This atypical E3 activity of HOIL1 diminishes the functions of LUBAC, which impacts various innate and adaptive immune signaling pathways (24, 30). It is possible that MALT1-dependent cleavage of HOIL1 may attenuate or regulate many of these functions.

Proteolytic cleavage of LUBAC has also emerged as an important regulatory mechanism (31). This discovery was empowered in part by the description of patients with homozygous germline pathogenic variants in MALT1 paracaspase, who presented with combined immunodeficiency, atopy, and features of autoinflammation such as inflammatory dermatitis and enteropathy (32–35). MALT1 is a protein that serves dual functions to promote signaling by acting as a scaffold to recruit additional signaling components, and functioning as a protease (specifically classified as a paracaspase) (36) to cleave known regulators of signaling (*e.g.* RelB, A20 and CYLD) (32). Leveraging MALT1-deficient patient cells, we defined a novel negative regulatory role for MALT1, whereby it cleaves HOIL1 to inhibit NF-κB activation (37). This HOIL1 cleavage event by MALT1 was also independently confirmed by other groups (38, 39), although the exact role of HOIL1 cleavage in regulating NF-κB activation and inflammatory responsiveness still remains unclear.

To further elucidate the impact of HOIL1 cleavage on NF-κB activation and inflammatory processes, in this study, we carefully defined the physiological relevance of MALT1-dependent cleavage of HOIL1. To do so we utilized skin fibroblasts from a unique patient who completely lacked detectable HOIL1 protein expression (16). This individual harbored compound heterozygous pathogenic variants in *RBCK1* which encodes HOIL1 (c.553 C>T, p.Q185X and 31.799Kb deletion of chromosome 20 del:*TRIB3*:g.-1272_*HOIL1*:g.9780del encompassing *TRIB3* and *HOIL1*) (16). We also previously showed that the conservative mutation of the cleavage site P1 arginine 165 (R165) to lysine (K) virtually abolished all MALT1-dependent cleavage of HOIL1 (37). Here we utilized this mutation to create a MALT1-resistant non-cleavable p.R165K variant of HOIL1 (henceforth HOIL1-R/K) that was stably expressed in primary skin fibroblasts from the HOIL1-deficient patient. This model system allowed us to specifically study the role of MALT1-dependent HOIL1 cleavage, while avoiding potential confounding effects from the cleavage of other MALT1 paracaspase substrates. The effect of HOIL1-R/K on LUBAC stability, NF-κB activation, the transcriptome, and cytokine secretion was assessed relative to wild-type HOIL1 (HOIL1-WT) transduced HOIL1-deficient fibroblasts. Here, we discovered that the inability of MALT1 paracaspase to cleave HOIL1 resulted in significantly enhanced canonical NF-κB activation, transcriptional enrichment of inflammatory pathways, and enhanced secretion of proinflammatory cytokines/chemokines in response to innate stimuli. Together these new findings reveal that MALT1-dependent cleavage of HOIL1 alone is critical for fine-tuning NF-κB signaling to limit inflammation and restore homeostasis.

## Results

### Construction and Characterization of Human Fibroblasts Expressing a MALT1 Paracaspase Resistant Non-Cleavable Version of HOIL1

**Figure 1:**
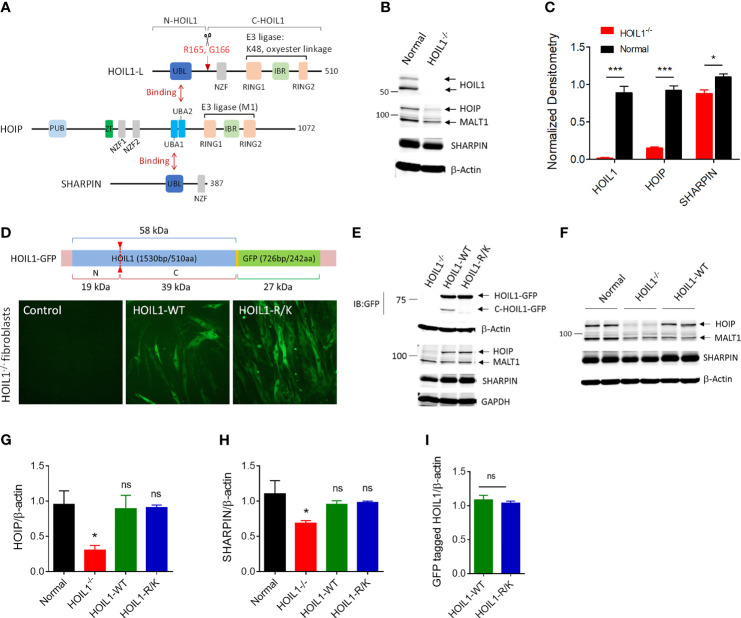
Construction and characterization of human cells expressing a MALT1-resistant non-cleavable HOIL1 (HOIL1-R/K). **(A)** A schematic diagram illustrating the structural domains of HOIL1-L, HOIP and SHARPIN forming the LUBAC. HOIL1-L, active isoform of HOIL1 in the LUBAC, is cleaved at R165 by MALT1 to produce N- and C-terminal fragments, N-HOIL1 and C-HOIL1, respectively. The UBL domain of HOIL1 binds to the UBA2 domain of HOIP, whereas the UBL domain of SHARPIN binds to the UBA1 domain of HOIP, forming a stable trimolecular complex. **(B)** Representative immunoblots showing the expression levels of HOIL1, HOIP, SHARPIN and MALT1 in control and HOIL1-deficient (HOIL1^–/–^) human fibroblasts. **(C)** Normalized (to β-actin) densitometry analyses of HOIL1, HOIP and SHARPIN protein levels from **(A)** (*N* ≥ 3). **(D)** Top: schematic representation of the HOIL1-GFP constructs used. Bottom: micrographs demonstrating transduction of GFP-tagged wild-type (WT) or non-cleavable (R/K) HOIL1 in HOIL1^–/–^ human fibroblasts. **(E)** Immunoblots showing transduction of different HOIL1-GFP constructs affecting HOIL1 cleavage capability and expression of HOIP, SHARPIN, and MALT1. **(F)** Immunoblots demonstrating the influence of HOIL1 expression on the stability of the other LUBAC components HOIP and SHARPIN compared to normal human fibroblasts. β-actin as the internal control. **(G, H)** Densitometry quantification of HOIP **(G)** and SHARPIN **(H)** levels in the four conditions: normal fibroblasts, HOIL1^–/–^ fibroblasts, HOIL1^–/–^ fibroblasts expressing HOIL1-WT and HOIL1^–/–^ fibroblasts expressing HOIL1-R/K (*N* ≥ 3). **(I)** Densitometry quantification of GFP-tagged HOIL1-WT and HOIL1-R/K expressed in HOIL1^–/–^ fibroblasts after transduction (*N* ≥ 3). *p < 0.05, ***p < 0.001, ns, non-significant relative to control unless otherwise indicated.

HOIL1 undergoes proteolytic cleavage by MALT1 after arginine-165 (R165) (**Figure 1A**) (37–39). To directly investigate the impact of this cleavage event on canonical NF-κB activation and whether inability to cleave HOIL1 could contribute to the clinical features of MALT1-deficient patients (32, 33, 35), we developed a non-cleavable version of HOIL1 by mutating R165 to lysine (designated HOIL1-R/K). In order to conduct these experiments in a human system while avoiding any contribution from endogenous HOIL1 protein, we obtained primary HOIL1-deficient (HOIL1^-/-^) fibroblasts derived from the skin of a patient with complete HOIL1 deficiency (16). This patient displayed clinical features of enhanced susceptibility to pyogenic bacterial infections, systemic autoinflammation (*i.e.* dermatitis, recurrent fevers and enteropathy) and amylopectinosis. We confirmed that HOIL1 deficiency had direct impact on the cellular stability of LUBAC (16, 17), where HOIP and SHARPIN expression was significantly decreased in the HOIL1-deficient fibroblasts (**Figures 1B, C**). We then used a lentiviral transduction approach to stably express GFP-tagged versions of HOIL1-R/K or wild-type HOIL1 (HOIL1-WT) in these cells (**Supplement Figure 1**). The expression of both HOIL1-WT and HOIL1-R/K restored the levels of HOIP and SHARPIN to levels indistinguishable from normal control cells (**Figures 1D–H**), and stabilized LUBAC expression (28). Importantly, the cells expressed equal amounts of GFP-tagged HOIL1-WT and HOIL1-R/K after transduction (**Figure 1I**) and the HOIL1-R/K could not be cleaved, as confirmed by the lack of a detectable C-HOIL1-GFP fragment by immunoblotting (**Figure 1E**).

### Evidence of Increased Canonical NF-κB Activation in the Presence of Non-Cleavable HOIL1

**Figure 2:**
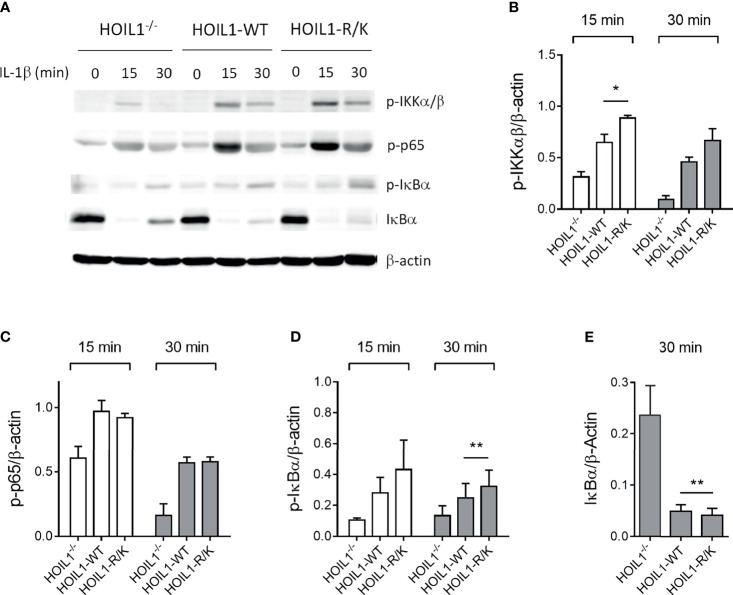
Non-cleavable HOIL1 leads to increased canonical NF-κB activation. **(A)** Activation of the canonical NF-κB pathway was assessed by stimulating HOIL1^–/–^, HOIL1-WT, HOIL1-R/K fibroblasts with 10 ng/mL IL-1β for 15 and 30 min and immunoblotting for phosphorylation of IKKα/β (p-IKKα/β), p65 (p-p65) and IκBα (p-IκBα), and degradation of IκBα. **(B-E)** Densitometry analyses of **(A)** for p-IKKα/β **(B)**, p-p65 **(C)**, p-IκBα **(D)** and IκBα degradation **(E)**. All panels are representative of at least 3 independent experiments. β-actin was used as an internal control. Paired t-tests were used to determine statistical significance between HOIL1-WT and HOIL1-R/K. *p < 0.05; **p < 0.01.

Since both HOIL1 and MALT1 have a clear role in mediating NF-κB activation (3) and MALT1 cleaves HOIL1 (37), we directly investigated the impact of MALT1 paracaspase-mediated cleavage of HOIL1 on the canonical NF-κB pathway using our model system. We stimulated HOIL1^–/–^, HOIL1-WT, and HOIL1-R/K fibroblasts with IL-1β across a time course and immunoblotted for key proteins in the canonical NF-κB pathway (**Figure 2** and **Supplementary Figure 2**). We demonstrated that expression of HOIL1-WT and HOIL1-R/K in HOIL1^–/–^ fibroblasts rescued canonical NF-κB activation as documented by the phosphorylation of the IKK complex (p-IKKα/β), the p65 subunit of NF-κB and (p-p65), and the inhibitor of NF-κB (p-IκBα), and the degradation of IκBα (**Figure 2A**). Furthermore, the presence of non-cleavable HOIL1 led to significantly enhanced canonical NF-κB activation. We performed statistical testing comparing the HOIL1-WT and HOIL1-R/K groups to assess if there was evidence of increased canonical NF-κB activation in the presence of non-cleavable HOIL1 and we found statistically significant differences for p-IKKα/β at 15 min (p = 0.024), p-IκBα at 30 min (p = 0.010), and IκBα at 30 min (p = 0.005) (**Figures 2B, D, E**).

### Presence of MALT1-Resistant HOIL1 Induces Global Changes in the Transcriptome Following Immune Stimulation

**Figure 3:**
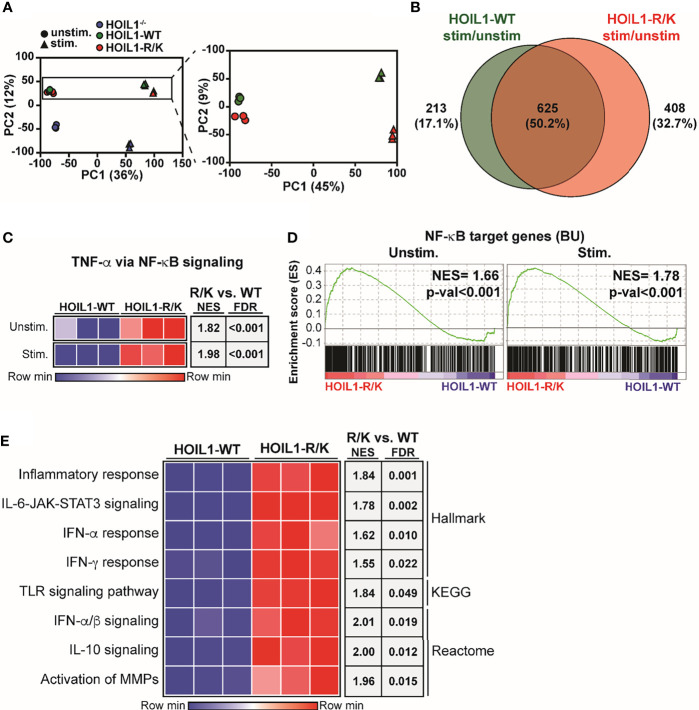
Impact of non-cleavable HOIL1 on the transcriptome. **(A)** Principal component analysis (PCA) comparing unstimulated and stimulated (10 ng/mL IL-1β for 4 h) HOIL1^–/–^ (blue), HOIL1-WT (green), HOIL1-R/K (red) fibroblasts. The contribution of PC1 or PC2 is shown in brackets. Triangles represent unstimulated and circles represent stimulated. Shown on the right is an inset of the HOIL1-WT and HOIL1-R/K region. **(B)** Venn diagram comparing number of genes that were significantly upregulated (adjusted p-val ≤ 0.05, fold change ≥ 1) in response to stimulation between HOIL1-WT and HOIL1-R/K. **(C)** Sample level enrichment analysis (SLEA) representation of the TNF-α *via* NF-κB signaling pathway (MSigDB Hallmark) in HOIL1-WT or HOIL1-R/K fibroblasts pre- and post-stimulation. The data in the heatmaps was scaled by the sum of each row. Shown on the right are normalized enrichment scores (NES) and false discovery rate (FDR) q-values of HOIL1-R/K vs. HOIL1-WT. **(D)** Gene set enrichment analysis plots of NF-κB target genes (The Gilmore Lab, Boston University [BU]) pre- or post-stimulation between HOIL1-R/K and HOIL1-WT. NES = normalized enrichment score. **(E)** SLEA representation of various major proinflammatory pathways. Shown on the right are NES and FDR q-val of stimulated HOIL1-R/K vs. stimulated HOIL1-WT.

Since we found biochemical evidence of enhanced canonical NF-κB pathway activation in the presence of non-cleavable HOIL1-R/K (**Figure 2**), we next examined whether this was associated with dysregulated gene expression. We profiled the global transcriptome by applying RNA-Seq to HOIL1^–/–^, HOIL1-WT transduced and HOIL1-R/K transduced fibroblasts with or without innate immune stimulation with IL-1β. Dimensionality reduction of transcriptomic data was achieved using the unsupervised learning algorithm principal component analysis (PCA). PCA showed that the unstimulated and stimulated conditions clustered separately (**Figure 3A**). As anticipated, the HOIL1^–/–^ fibroblasts clustered entirely separately from the HOIL1-WT and HOIL1-R/K fibroblasts in both conditions (**Figure 3A**, left panel). Specifically comparing the transcriptome of HOIL1-WT and HOIL1-R/K fibroblasts revealed that HOIL1-WT and HOIL1-R/K clustered separately on PC2 and these differences were exaggerated following immune stimulation (**Figure 3A**, right panel). Furthermore, we found evidence of dysregulated gene expression in the presence of non-cleavable HOIL1. When examining differentially expressed genes significantly upregulated (fold change [FC] ≥ 1, adjusted p-val ≤ 0.05) in response to IL-1β stimulation between HOIL1-WT and HOIL1-R/K, we discovered that although 625 genes (50.2%) were commonly upregulated regardless of the HOIL1 variant, 408 genes (32.7%) were exclusively upregulated in the presence of HOIL1-R/K vs. 213 (17.1%) with HOIL1-WT 4 h after IL-1β stimulation (**Figure 3B**). This pattern also held true for genes that were significantly downregulated (FC ≤ 1, adjusted p-val ≤ 0.05), with HOIL1-R/K associated with exclusively downregulating 897 genes (51.3%) vs. 414 (23.7%) with HOIL1-WT (**Supplementary Figure 3**).

### MALT1-Dependent Cleavage of HOIL1 Dampens NF-κB Target Gene Expression and Major Proinflammatory Pathways

Since HOIL1-R/K fibroblasts showed significant global transcriptomic differences from HOIL1-WT fibroblasts following immune stimulation (**Figures 3A, B**), we sought to define the pathways that were dysregulated. Confirming our biochemical data (**Figure 2**), gene set enrichment analysis (GSEA) considering normalized enrichment scores (NES) and false discovery rate (FDR) demonstrated that fibroblasts expressing non-cleavable HOIL1-R/K were significantly enriched in the ‘TNF-α *via* NF-κB signaling’ pathway both at baseline (NES = 1.82, FDR < 0.001) and in response to IL-1β stimulation (NES = 1.98, FDR < 0.001), relative to HOIL1-WT fibroblasts (**Figure 3C**). A similar result was observed when we extended this analysis by creating a gene set of all annotated NF-κB target genes (40) (here designated BU), with significant enrichment at baseline (NES = 1.66, p-val < 0.001) and in response to IL-1β stimulation (NES = 1.78, p-val < 0.001) (**Figure 3D**). In addition to the NF-κB pathway, stimulated HOIL1-R/K fibroblasts were significantly more enriched in curated pathways involved in inflammation, cytokine signaling, and innate immunity. This includes the inflammatory response (NES = 1.84, FDR = 0.001), IL-6-JAK-STAT3 signaling (NES = 1.78, FDR = 0.002), IFN-α/β signaling (NES = 2.01, FDR = 0.019), IFN-α response (NES = 1.62, FDR = 0.010), IFN-γ response (NES = 1.55, FDR = 0.022), TLR signaling pathway (NES = 1.84, FDR = 0.049), IL-10 signaling (NES = 2.00, FDR = 0.012), and activation of matrix metalloproteinases (MMPs) (NES = 1.96, FDR = 0.015) (**Figure 3E**).

### Proinflammatory Cytokine and Chemokine Production Is Increased in the Presence of Non-Cleavable HOIL1

**Figure 4:**
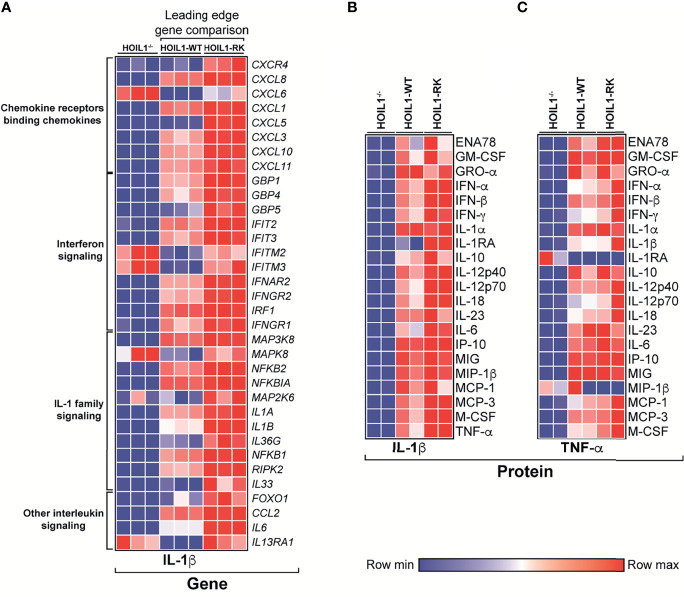
Non-cleavable HOIL1 promotes exaggerated proinflammatory cytokine and chemokine production. **(A)** Heatmap representation of normalized counts of leading edge (LE) genes obtained by GSEA of major cytokine/chemokine-related pathways (Reactome) comparing stimulated HOIL1-R/K vs. HOIL1-WT. Normalized counts for HOIL1^–/–^ were included for qualitative comparison. **(B, C)** Heatmap representation of cytokine concentrations 24 h after IL-1β **(B)** or TNF-α **(C)** stimulation of HOIL1^–/–^, HOIL1-WT and HOIL1-R/K fibroblasts as measured by 23-plex multiplexed cytokine assays (*N* = 2). All heatmaps were row normalized.

Since the presence of a non-cleavable version of HOIL1 resulted in significant enrichment in the transcription of a number of cytokine/chemokine-related pathways (**Figure 3E**), we specifically compared the transcript abundance of leading edge ‘driver’ genes in these pathways between HOIL1-RK and HOIL1-WT, with HOIL1^–/–^ fibroblasts serving as an additional control (**Figure 4A**). While the rescue of HOIL1 expression (HOIL1-WT) in HOIL1^–/–^ fibroblasts restored the upregulation of many of these leading-edge cytokine/chemokine genes, HOIL1-R/K resulted in greater increases in abundance of these same genes (**Figure 4A**). To assess whether these profound transcriptional changes translated to exaggerated downstream effector functions (*i.e.* cytokine/chemokine secretion), we stimulated HOIL1^–/–^, HOIL1-WT, and HOIL1-R/K fibroblasts with IL-1β or TNF-α and measured the secretion of a panel of 23 proinflammatory cytokines/chemokines (**Figures 4B, C**). Recapitulating our transcriptome and ELISA data (**Supplementary Figure 4**), the secretion of the vast majority of these cytokines/chemokines were elevated in the HOIL1-R/K fibroblasts in response to both IL-1β and TNF-α stimulation, relative to HOIL1-WT and HOIL1^–/–^ fibroblasts (**Figures 4B, C**).

## Discussion

MALT1 paracaspase was recently identified as a novel negative regulator of LUBAC-mediated signaling, whereby it cleaves HOIL1 to destabilize LUBAC assembly (37–39). As MALT1 has 12 substrates reported to date, and each regulates NF-κB or other signaling pathways differently (41–43), it is difficult to study the specific effects of cleavage of individual substrates by simply inhibiting MALT1 protease activity or genetically knocking-out/down MALT1. To directly investigate the role of MALT1-dependent cleavage of HOIL1 in immunity and physiology, we generated a non-cleavable version of HOIL1 (HOIL1-R/K) and stably expressed this construct in primary HOIL1-deficient patient fibroblasts. We established that expressing HOIL1-R/K resulted in an exaggerated inflammatory response to innate stimuli, characterized by enhanced activation of the canonical NF-κB pathway, leading to enhanced proinflammatory cytokine/chemokine gene and protein expression.

While this work and our experimental systems have many strengths, we do acknowledge that there are some weaknesses. For example, we did not experimentally establish that the reconstituted HOIL1-WT-GFP and HOIL1-R/K-GFP do bind to the endogenous HOIP and SHARPIN to form the LUBAC complex. However, many lines of experimental and literature evidence support their interaction in our cellular model including the stabilization of endogenous HOIP and SHARPIN expression (**Figures 1E–H**) (16, 17, 19, 28) and the restoration of NF-κB signaling (**Figure 2**) (16, 17, 19). We also acknowledge that the transcriptomic differences observed in the presence of non-cleavable HOIL1-R/K could be caused by kinetic changes in a variety of the genes that we identified to be significantly different between HOIL1-WT and HOIL1-R/K. Future work will focus on identifying how these genes change over time.

Empowered by increasing availability of next-generation sequencing technology, more patients with monogenic immune disorders are being identified (44). When considered in the context of other experimental model systems such as genetically engineered mice, these patients provide powerful insights into fundamental human biology. Patients with defects in MALT1, LUBAC and OTULIN all share a common feature of exaggerated inflammation (16–22). However, the mechanisms underlying this autoinflammation are clearly nuanced as mice engineered to lack only MALT1 paracaspase activity develop multiorgan inflammation (45–47).

**Figure 5:**
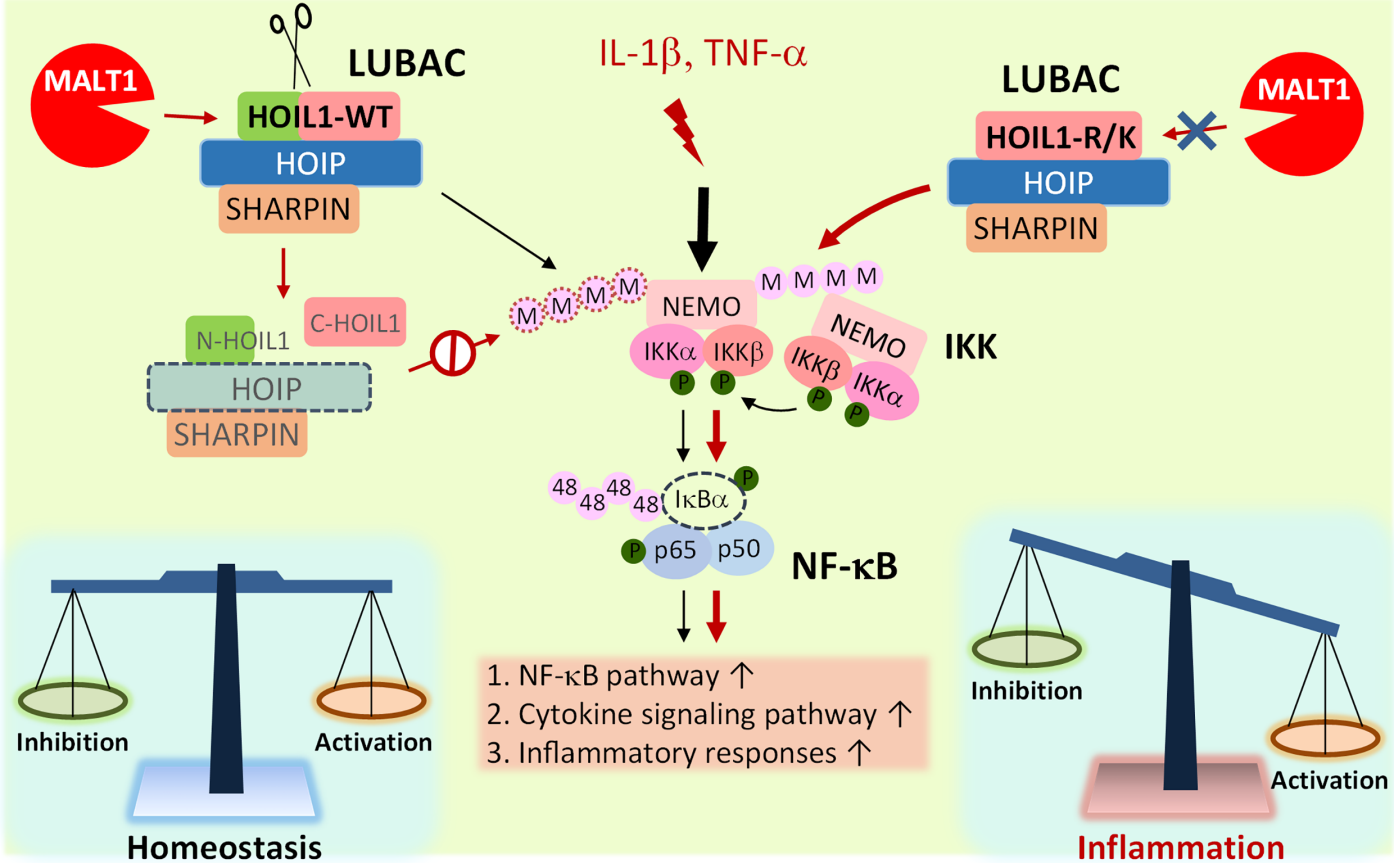
Model of MALT1-mediated cleavage of HOIL1 and subsequent regulation of NF-κB and inflammation. Innate stimuli such as IL-1β and TNF-α trigger LUBAC assembly and linear ubiquitination of various proteins such as NEMO (IKKγ) to promote the canonical activation of NF-κB. Over time, when a specific signaling threshold has been reached, MALT1 proteolytically cleaves HOIL1 to disassemble LUBAC and decrease its activity to fine-tune NF-κB activation and restore homeostasis. In the absence of MALT1-dependent proteolytic regulation, as is the case in HOIL1-R/K and MALT1-deficient patients, NF-κB activation is sustained, leading to a shift towards a more proinflammatory phenotype in certain cells.

Based on our experimental evidence and informed by clinical experience with MALT1-deficient patients, we propose a model whereby MALT1-dependent cleavage of HOIL1 is a major link in the negative feedback chain to dampen NF-κB activation after initial NF-κB stimulation in order to modulate the inflammatory response and avoid excessive inflammation and associated pathology (**Figure 5**). Upon inflammatory stimulation by IL-1β or TNF-α, the LUBAC promotes NF-κB activation and proinflammatory gene and protein expression. After initial NF-κB activation, MALT1-mediated cleavage of HOIL1 destabilizes the LUBAC and dampens NF-κB signaling, serving as a regulatory signal to avoid exaggerated activation. When HOIL1 is unable to be cleaved, the balance between pro-NF-κB and anti-NF-κB activation is disrupted, as is the case with MALT1-deficient patients or MALT1 protease dead mice, leading to sustained NF-κB activation and subsequent pathological proinflammatory responses. While this theoretical model incorporates much of our current understanding of biology, it is likely incomplete. It is currently unknown how MALT1-mediated cleavage of HOIL1 impacts recently discovered roles for HOIL1, including the ability to conjugate monoubiquitin onto all LUBAC subunits (24) and ester bond formation between ubiquitin and serine and threonine residues in its substrates (29).

MALT1 paracaspase inhibitors are currently under intense study as potential therapeutics against lymphomas (48–51), multiple sclerosis (52), colitis (46), and psoriatic skin diseases (53). This study highlights the physiological importance of MALT1-dependent cleavage and modulation of HOIL1 on NF-κB signaling and inflammation, provides a mechanism for the autoinflammation observed in MALT1-deficient patients, and will inform the development of therapeutics that target MALT1 paracaspase and LUBAC function. In particular, this current study is a reminder that long-term inhibition of MALT1 paracaspase activity may increase the risk of exaggerated inflammation in certain cell types.

## Materials and Methods

### Study Participants and Ethics Approvals

Research protocols were approved by the University of British Columbia and Children’s and Women’s Health Center of British Columbia Research Ethics Board. Simian virus 40 (SV40)-transformed human fibroblasts derived from a skin biopsy of a HOIL1-deficient patient (16) were obtained through a material transfer agreement from Dr. Luigi D. Notarangelo (National Institutes of Health, Bethesda, MD, USA), with permission for research use.

### Stable Expression of Non-Cleavable HOIL1

MALT1 cleaves HOIL1 after arginine (R) at position 165 (LxS/PR) (37). Using site-directed mutagenesis (Q5, New England BioLabs), R165 was mutated to lysine (K) (R/K) on a wild-type (WT) HOIL1 plasmid (#RC203906, OriGene Technologies) to maintain charge at this location (**Supplementary Figure 1**). The long isoform transcript variant 2 of HOIL1 (also known as HOIL1-L) was selected for our study as it is the main and active form of HOIL1 in the LUBAC (9). Both HOIL1-WT and HOIL1-R/K were cloned into a GFP-tagged Lenti vector (#PS100071, OriGene Technologies), confirmed by sequencing of the vectors (**Supplementary Table 1**), and packaged using a Lenti-vpack packaging kit (#TR30037, OriGene Technologies) in HEK293T cells according to the manufacturer’s instructions. Briefly, culture media was collected, centrifuged, filtered, concentrated, and stored at -80°C before use. The viral titer was determined on HEK293T cells through serial dilution. To establish fibroblasts stably expressing HOIL1-WT or HOIL1-R/K, HOIL1^–/–^ fibroblasts were infected with HOIL1-WT or HOIL1-R/K lentiviral particles at a ratio of 0.5:1. All fibroblasts were cultured and expanded in high glucose DMEM (HyClone) supplemented with 10% FBS and 2 mM L-glutamine. Expanded cells were sorted on GFP expression (BD FACS Aria IIu, BD Biosciences).

### Stimulations

For signaling analyses, HOIL1^–/–^, HOIL1-WT, or HOIL1-R/K fibroblasts were stimulated with 10 ng/mL IL-1β (PeproTech, USA) for 15 or 30 min. Transcriptome analyses were carried out 4 h after 10 ng/mL IL-1β stimulation. To analyze cytokine secretion by multiplexed cytokine assays, fibroblasts were stimulated with 10 ng/mL IL-1β or 20 ng/mL TNF-α (eBioscience, Thermo Fisher Scientific) for 24 h.

### Immunoblotting for Signaling and Protein Detection

Immunoblotting was performed as previously described (37, 54). Briefly, cells were lysed in a modified RIPA lysis buffer (50 mM Tris-HCl, 150 mM NaCl, 2 mM EGTA and EDTA, 1% Triton X-100, pH 7.5, Sigma-Aldrich) supplemented with 1x HALT protease and phosphatase inhibitors (Thermo Fisher Scientific). Lysates were separated by 10% SDS-PAGE and transferred onto PVDF membranes (0.45 μm, Immobilon-FL, EMD Millipore). Membranes were blocked, incubated with primary antibodies, washed, incubated with secondary antibodies, and imaged on a LI-COR Odyssey infrared imager (LI-COR Biosciences). Bands were quantified by densitometry using ImageJ freeware (NIH). The following primary antibodies were used: phospho-IKKα/β (Ser176/180, #2697), phospho-NF-κB p65 (Ser536, #3031), IκBα (#9492), phospho-IκBα (#9246), GFP (#2956) and β-actin (#3700 or #8457) all from Cell Signaling Technology; C-terminal HOIL1 (2E2) and GAPDH (6C5) were from Millipore Sigma; MALT1 (EP603Y), HOIP (ab46322) and SHARPIN (ab197853) were from Abcam. The following secondary antibodies were used: DyLight 800 (#611-145-002, Rockland Immunochemicals) and IRDye 680RD (#926-68070, LI-COR Biosciences).

### Multiplexed Cytokine Analysis

Supernatants from each condition (*N =* 2) were collected by centrifugation and diluted in 96-well assay plates. Multiplexed cytokine analyses were performed using a Luminex-based Procarta custom 23-Plex assay (eBioscience) according to manufacturer’s recommendations. The 23 cytokines quantified in the assay included: ENA78 (CXCL5), GM-CSF, GRO-α (CXCL1), IFN-α, IFN-β, IFN-γ, IL-1α, IL-1β, IL-1RA, IL-10, IL-12p40, IL-12p70, IL-18, IL-23, IL-6, IP-10 (CXCL10), MIG (CXCL9), MIP-1β (CCL4), MCP-1 (CCL2), MCP-3 (CCL7), M-CSF and IL-8 (CXCL8). Assays were read on a Luminex 200 Total System with MasterPlex (MiraiBio) software. Mean fluorescence intensity of the samples was interpolated to analyte quantity using five-parameter logistical fit. IL-8 was excluded as it was above the limit of detection. TNF-α and IL-1β values were excluded in the TNF-α and IL-1β stimulation conditions, respectively.

### RNA-Seq and Transcriptomic Analyses

RNA was extracted from HOIL1^–/–^, HOIL1-WT, HOIL1-R/K fibroblasts with or without stimulation in triplicate using a RNeasy Mini kit (QIAGEN). Quality control was performed using an Agilent 2100 Bioanalyzer (Agilent Technologies); all samples had RIN ≥ 9.7. Samples were prepared according to the standard protocol for the NEBNext Ultra II Stranded mRNA (New England BioLabs), sequenced on an Illumina NextSeq 500 with Paired End reads (42 bp x 42 bp), and analyzed using standard bioinformatics methods in the following.

De-multiplexed read sequences were aligned to a reference sequence using STAR aligner (55). Assembly and differential expression was estimated using Cufflinks (56) on bioinformatics apps on Illumina BaseSpace. Expression was normalized to total read depth between samples using the edgeR package in R. Principal component analysis (PCA) was done on log_2_(normalized counts+0.25) in R using the PCA function. PCA was performed on genes that had been filtered for non-zero counts across replicates.

To determine genes that were significantly upregulated or downregulated, the fold change between stimulated and unstimulated was determined for HOIL1-WT or HOIL1-R/K and t-tests were performed. This was carried out on Trimmed Mean of M-values (TMM) normalized counts. The p-values were adjusted for multiple comparisons using the Benjamini-Hochberg (BH) method. Venn diagram comparisons were done using Venny (https://bioinfogp.cnb.csic.es/tools/venny/).

Pathway analysis was accomplished by gene set enrichment analyses (GSEA) using MSigDB’s Hallmark module, KEGG, and Reactome for each pathway. GSEA was also carried out on 462 NF-κB target genes obtained from Boston University (https://www.bu.edu/nf-kb/gene-resources/target-genes/). Signal-to-noise was used for gene ranking and the obtained normalized enrichment scores and p-values were further adjusted using the BH method. Pathways with an adjusted p-value < 0.05 were considered significant. Leading edge genes from each pathway were obtained for HOIL1-R/K vs. HOIL1-WT. The expression levels of these genes were then determined in each group.

Sample level enrichment analyses (SLEA) scores were computed as previously described (57). The mean expression value was calculated for the gene set of interest and was compared to the expression of 1000 random gene sets of the same size. The difference between observed and expected mean expression was then calculated, and the z-score was plotted on heatmaps as the SLEA scores. All heatmaps were generated using Morpheus (https://software.broadinstitute.org/morpheus).

### Statistical Analysis

All data were presented as means ± standard errors of the means (SEM). Statistical significance was evaluated by one-way ANOVA with the Bonferroni post-test (whenever applicable), except for **Figure 2** where a paired t-test was performed comparing the HOIL1-WT and HOIL1-R/K groups. The following annotations were used to represent significance: p-val < 0.05 (*), p-val < 0.01 (**), p-val < 0.001 (***).

## Data Availability Statement

The original contributions presented in the study are publicly available. This data can be found here: https://www.ncbi.nlm.nih.gov/geo/query/acc.cgi?acc=GSE181493.

## Ethics Statement

The studies involving human participants were reviewed and approved by The University of British Columbia and Children’s and Women’s Health Center of British Columbia Research Ethics Board. Written informed consent to participate in this study was provided by the participants’ legal guardian/next of kin.

## Author Contributions

SYF, ST, CO, TK, and MG conceived the study and designed the research. SYF and HL wrote the manuscript, analyzed data, and made figures with input from MS, AAS, AJ, LA, AS, MG, and ST. SYF. established the non-cleavable HOIL1 transduction system, performed the immunoblotting experiments, multiplexed cytokine assays, and sample preparation for RNA-Seq using unique reagents from LDN. HL, MS, AAS, and AS performed RNA-Seq data analysis. HL, AJ, and LA performed technical replicates for experiments. All authors revised and approved the final version of the manuscript.

## Funding

This study was supported by grants from Canadian Institutes of Health Research (CIHR) (ST), Genome British Columbia (SIP007) (ST), National Natural Science Foundation of China (No. 81971549 for SYF), the Canadian Allergy, Asthma and Immunology Foundation, and BC Children’s Hospital Foundation. ST holds the Tier 1 Canada Research Chair in Pediatric Precision Health and the Aubrey J. Tingle Professorship in Pediatric Immunology. LN is supported by the Division of Intramural Research, National Institute of Allergy and Infectious Diseases, National Institutes of Health. HL is funded by a CIHR Frederick Banting and Charles Best Canada Graduate Scholarship (CGS-D), Killam Doctoral Scholarship, University of British Columbia Four Year Doctoral Fellowship (4YF), and a BC Children’s Hospital Research Institute Graduate Studentship. MS is funded by a CIHR CGS-D and 4YF. AJ is funded by a CIHR Canada Graduate Scholarship-Master’s (CGS-M). CO is supported by a CIHR Foundation Grant (FDN148408) and a Canada Research Chair in Protease Proteomics and Systems Biology.

## Conflict of Interest

TK was employed by Ducares/Triskelion BV.

The remaining authors declare that the research was conducted in the absence of any commercial or financial relationships that could be construed as a potential conflict of interest.

## Publisher’s Note

All claims expressed in this article are solely those of the authors and do not necessarily represent those of their affiliated organizations, or those of the publisher, the editors and the reviewers. Any product that may be evaluated in this article, or claim that may be made by its manufacturer, is not guaranteed or endorsed by the publisher.

## References

[1] Lawrence T. The Nuclear Factor NF-kappaB Pathway in Inflammation. Cold Spring Harb Perspect Biol (2009) 1(6):a001651. doi: 10.1101/cshperspect.a001651 20457564 PMC2882124

[2] Taniguchi K Karin M. NF-Kappab, Inflammation, Immunity and Cancer: Coming of Age. Nat Rev Immunol (2018) 18(5):309–24. doi: 10.1038/nri.2017.142 29379212

[3] Zhang Q Lenardo MJ Baltimore D. 30 Years of NF-Kappab: A Blossoming of Relevance to Human Pathobiology. Cell (2017) 168(1-2):37–57. doi: 10.1016/j.cell.2016.12.012 28086098 PMC5268070

[4] Sun SC. The Non-Canonical NF-KappaB Pathway in Immunity and Inflammation. Nat Rev Immunol (2017) 17(9):545–58. doi: 10.1038/nri.2017.52 PMC5753586 28580957

[5] Aksentijevich I Zhou Q. NF-kappaB Pathway in Autoinflammatory Diseases: Dysregulation of Protein Modifications by Ubiquitin Defines a New Category of Autoinflammatory Diseases. Front Immunol (2017) 8:399. doi: 10.3389/fimmu.2017.00399 28469620 PMC5395695

[6] Liu T Zhang L Joo D Sun SC. NF-kappaB Signaling in Inflammation. Signal Transduct Target Ther (2017) 2:17023. doi: 10.1038/sigtrans.2017.23 29158945 PMC5661633

[7] Oeckinghaus A Ghosh S. The NF-KappaB Family of Transcription Factors and Its Regulation. Cold Spring Harb Perspect Biol (2009) 1(4):a000034. doi: 10.1101/cshperspect.a000034 20066092 PMC2773619

[8] Hu H Sun SC. Ubiquitin Signaling in Immune Responses. Cell Res (2016) 26(4):457–83. doi: 10.1038/cr.2016.40 PMC4822134 27012466

[9] Kirisako T Kamei K Murata S Kato M Fukumoto H Kanie M. A Ubiquitin Ligase Complex Assembles Linear Polyubiquitin Chains. EMBO J (2006) 25(20):4877–87. doi: 10.1038/sj.emboj.7601360 PMC1618115 17006537

[10] Ikeda F Deribe YL Skanland SS Stieglitz B Grabbe C Franz-Wachtel M. SHARPIN Forms a Linear Ubiquitin Ligase Complex Regulating NF-kappaB Activity and Apoptosis. Nature (2011) 471(7340):637–41. doi: 10.1038/nature09814 PMC3085511 21455181

[11] Haas TL Emmerich CH Gerlach B Schmukle AC Cordier SM Rieser E. Recruitment of the Linear Ubiquitin Chain Assembly Complex Stabilizes the TNF-R1 Signaling Complex and Is Required for TNF-Mediated Gene Induction. Mol Cell (2009) 36(5):831–44. doi: 10.1016/j.molcel.2009.10.013 20005846

[12] Tokunaga F Sakata S Saeki Y Satomi Y Kirisako T Kamei K. Involvement of Linear Polyubiquitylation of NEMO in NF-KappaB Activation. Nat Cell Biol (2009) 11(2):123–32. doi: 10.1038/ncb1821 19136968

[13] Komander D Reyes-Turcu F Licchesi JD Odenwaelder P Wilkinson KD Barford D. Molecular Discrimination of Structurally Equivalent Lys 63-Linked and Linear Polyubiquitin Chains. EMBO Rep (2009) 10(5):466–73. doi: 10.1038/embor.2009.55 PMC2680876 19373254

[14] Rahighi S Ikeda F Kawasaki M Akutsu M Suzuki N Kato R. Specific Recognition of Linear Ubiquitin Chains by NEMO Is Important for NF-KappaB Activation. Cell (2009) 136(6):1098–109. doi: 10.1016/j.cell.2009.03.007 19303852

[15] Sasaki K Iwai K. Roles of Linear Ubiquitinylation, a Crucial Regulator of NF-kappaB and Cell Death, in the Immune System. Immunol Rev (2015) 266(1):175–89. doi: 10.1111/imr.12308 26085215

[16] Boisson B Laplantine E Prando C Giliani S Israelsson E Xu Z. Immunodeficiency, Autoinflammation and Amylopectinosis in Humans With Inherited HOIL-1 and LUBAC Deficiency. Nat Immunol (2012) 13(12):1178–86. doi: 10.1038/ni.2457 PMC3514453 23104095

[17] Boisson B Laplantine E Dobbs K Cobat A Tarantino N Hazen M. Human HOIP and LUBAC Deficiency Underlies Autoinflammation, Immunodeficiency, Amylopectinosis, and Lymphangiectasia. J Exp Med (2015) 212(6):939–51. doi: 10.1084/jem.20141130 PMC4451137 26008899

[18] Boisson B Casanova JL. LUBAC: A New Function in Immunity. J Exp Med (2014) 211(7):1272. doi: 10.1084/jem.2117insight3 24980746 PMC4076582

[19] Oda H Beck DB Kuehn HS Sampaio Moura N Hoffmann P Ibarra M. Second Case of HOIP Difficiency Expands Clinical Features and Defines Inflammatory Transcriptome Regulated by LUBAC. Front Immunol (2019) 10:479. doi: 10.3389/fimmu.2019.00479 30936877 PMC6431612

[20] Zhou Q Yu X Demirkaya E Deuitch N Stone D Tsai WL. Biallelic Hypomorphic Mutations in a Linear Deubiquitinase Define Otulipenia, an Early-Onset Autoinflammatory Disease. Proc Natl Acad Sci USA (2016) 113(36):10127–32. doi: 10.1073/pnas.1612594113 PMC5018768 27559085

[21] Damgaard RB Walker JA Marco-Casanova P Morgan NV Titheradge HL Elliott PR. The Deubiquitinase OTULIN Is an Essential Negative Regulator of Inflammation and Autoimmunity. Cell (2016) 166(5):1215–30.e1220. doi: 10.1016/j.cell.2016.07.019 27523608 PMC5002269

[22] Nabavi M Shahrooei M Rokni-Zadeh H Vrancken J Changi-Ashtiani M Darabi K. Auto-Inflammation in a Patient With a Novel Homozygous OTULIN Mutation. J Clin Immunol (2019) 39(2):138–41. doi: 10.1007/s10875-019-00599-3 30796585

[23] Taraborrelli L Peltzer N Montinaro A Kupka S Rieser E Hartwig T. LUBAC Prevents Lethal Dermatitis by Inhibiting Cell Death Induced by TNF, TRAIL and CD95L. Nat Commun (2018) 9(1):3910. doi: 10.1038/s41467-018-06155-8 30254289 PMC6156229

[24] Fuseya Y Fujita H Kim M Ohtake F Nishide A Sasaki K. The HOIL-1L Ligase Modulates Immune Signalling and Cell Death via Monoubiquitination of LUBAC. Nat Cell Biol (2020) 22(6):663–73. doi: 10.1038/s41556-020-0517-9 32393887

[25] Zinngrebe J Rieser E Taraborrelli L Peltzer N Hartwig T Ren H. –LUBAC Deficiency Perturbs TLR3 Signaling to Cause Immunodeficiency and Autoinflammation. J Exp Med (2016) 213(12):2671–89. doi: 10.1084/jem.20160041 PMC5110014 27810922

[26] Hrdinka M Fiil BK Zucca M Leske D Bagola K Yabal M. CYLD Limits Lys63- and Met1-Linked Ubiquitin at Receptor Complexes to Regulate Innate Immune Signaling. Cell Rep (2016) 14(12):2846–58. doi: 10.1016/j.celrep.2016.02.062 PMC4819907 26997266

[27] Keusekotten K Elliott PR Glockner L Fiil BK Damgaard RB Kulathu Y. OTULIN Antagonizes LUBAC Signaling by Specifically Hydrolyzing Met1-Linked Polyubiquitin. Cell (2013) 153(6):1312–26. doi: 10.1016/j.cell.2013.05.014 PMC3690481 23746843

[28] Fujita H Tokunaga A Shimizu S Whiting AL Aguilar-Alonso F Takagi K. Cooperative Domain Formation by Homologous Motifs in HOIL-1L and SHARPIN Plays a Crucial Role in LUBAC Stabilization. Cell Rep (2018) 23(4):1192–204. doi: 10.1016/j.celrep.2018.03.112 PMC6044281 29694895

[29] Kelsall IR Zhang J Knebel A Arthur JSC Cohen P. The E3 Ligase HOIL-1 Catalyses Ester Bond Formation Between Ubiquitin and Components of the Myddosome in Mammalian Cells. Proc Natl Acad Sci USA (2019) 116(27):13293–8. doi: 10.1073/pnas.1905873116 PMC6613137 31209050

[30] Petrova T Zhang J Nanda SK Figueras-Vadillo C Cohen P. HOIL-1-Catalysed Ester-Linked Ubiquitylation Restricts IL-18 Signaling in Cytotoxic T Cells But Promotes TLR Signalling in Macrophages. FEBS J (2021). doi: 10.1111/febs.15896 33932090

[31] Hrdinka M Gyrd-Hansen M. The Met1-Linked Ubiquitin Machinery: Emerging Themes of (De)Regulation. Mol Cell (2017) 68(2):265–80. doi: 10.1016/j.molcel.2017.09.001 29053955

[32] Lu HY Bauman BM Arjunaraja S Dorjbal B Milner JD Snow AL. The CBM-Opathies-a Rapidly Expanding Spectrum of Human Inborn Errors of Immunity Caused by Mutations in the CARD11-BCL10-MALT1 Complex. Front Immunol (2018) 9:2078. doi: 10.3389/fimmu.2018.02078 30283440 PMC6156466

[33] Lu HY Biggs CM Blanchard-Rohner G Fung SY Sharma M Turvey SE. Germline CBM-Opathies: From Immunodeficiency to Atopy. J Allergy Clin Immunol (2019) 143(5):1661–73. doi: 10.1016/j.jaci.2019.03.009 31060714

[34] McKinnon ML Rozmus J Fung SY Hirschfeld AF Del Bel KL Thomas L. Combined Immunodeficiency Associated With Homozygous MALT1 Mutations. J Allergy Clin Immunol (2014) 133(5):1458–62, 1462.e1451–7. doi: 10.1016/j.jaci.2013.10.045 24332264

[35] Lu HY Turvey SE. Human MALT1 Deficiency and Predisposition to Infections. Curr Opin Immunol (2021) 72:1–12. doi: 10.1016/j.coi.2021.02.008 33714841

[36] Minina EA Staal J Alvarez VE Berges JA Berman-Frank I Beyaert R. Classification and Nomenclature of Metacaspases and Paracaspases: No More Confusion With Caspases. Mol Cell (2020) 77(5):927–9. doi: 10.1016/j.molcel.2019.12.020 PMC7325697 32142688

[37] Klein T Fung SY Renner F Blank MA Dufour A Kang S. The Paracaspase MALT1 Cleaves HOIL1 Reducing Linear Ubiquitination by LUBAC to Dampen Lymphocyte NF-KappaB Signalling. Nat Commun (2015) 6:8777. doi: 10.1038/ncomms9777 26525107 PMC4659944

[38] Elton L Carpentier I Staal J Driege Y Haegman M Beyaert R. MALT1 Cleaves the E3 Ubiquitin Ligase HOIL-1 in Activated T Cells, Generating a Dominant Negative Inhibitor of LUBAC-Induced NF-kappaB Signaling. FEBS J (2016) 283(3):403–12. doi: 10.1111/febs.13597 26573773

[39] Douanne T Gavard J Bidere N. The Paracaspase MALT1 Cleaves the LUBAC Subunit HOIL1 During Antigen Receptor Signaling. J Cell Sci (2016) 129(9):1775–80. doi: 10.1242/jcs.185025 27006117

[40] Boston U. NF-kB Target Genes Online (2020). Available at: https://www.bu.edu/nf-kb/gene-resources/target-genes/ (Accessed June 2020).

[41] Ruland J Hartjes L. CARD-BCL-10-MALT1 Signalling in Protective and Pathological Immunity. Nat Rev Immunol (2019) 19(2):118–34. doi: 10.1038/s41577-018-0087-2 30467369

[42] Israel L Gluck A Berger M Coral M Ceci M Unterreiner A. CARD10 Cleavage by MALT1 Restricts Lung Carcinoma Growth. Vivo Oncogene (2021) 10(4):32. doi: 10.1038/s41389-021-00321-2 PMC8024357 33824280

[43] Yamasoba D Sato K Ichinose T Imamura T Koepke L Joas S. N4BP1 Restricts HIV-1 and Its Inactivation by MALT1 Promotes Viral Reactivation. Nat Microbiol (2019) 4(9):1532–44. doi: 10.1038/s41564-019-0460-3 31133753

[44] Tangye SG Al-Herz W Bousfiha A Chatila T Cunningham-Rundles C Etzioni A. Human Inborn Errors of Immunity: 2019 Update on the Classification From the International Union of Immunological Societies Expert Committee. J Clin Immunol (2020) 40(1):24–64. doi: 10.1007/s10875-019-00737-x 31953710 PMC7082301

[45] Gewies A Gorka O Bergmann H Pechloff K Petermann F Jeltsch KM. Uncoupling Malt1 Threshold Function From Paracaspase Activity Results in Destructive Autoimmune Inflammation. Cell Rep (2014) 9(4):1292–305. doi: 10.1016/j.celrep.2014.10.044 25456129

[46] Jaworski M Marsland BJ Gehrig J Held W Favre S Luther SA. Malt1 Protease Inactivation Efficiently Dampens Immune Responses But Causes Spontaneous Autoimmunity. EMBO J (2014) 33(23):2765–81. doi: 10.15252/embj.201488987 PMC4282555 25319413

[47] Bornancin F Renner F Touil R Sic H Kolb Y Touil-Allaoui I. Deficiency of MALT1 Paracaspase Activity Results in Unbalanced Regulatory and Effector T and B Cell Responses Leading to Multiorgan Inflammation. J Immunol (2015) 194(8):3723–34. doi: 10.4049/jimmunol.1402254 25762782

[48] Fontan L Qiao Q Hatcher JM Casalena G Us I Teater M. Specific Covalent Inhibition of MALT1 Paracaspase Suppresses B Cell Lymphoma Growth. J Clin Invest (2018) 128(10):4397–412. doi: 10.1172/JCI99436 PMC6159983 30024860

[49] Nagel D Spranger S Vincendeau M Grau M Raffegerst S Kloo B. Pharmacologic Inhibition of MALT1 Protease by Phenothiazines as a Therapeutic Approach for the Treatment of Aggressive ABC-DLBCL. Cancer Cell (2012) 22(6):825–37. doi: 10.1016/j.ccr.2012.11.002 23238017

[50] Lim SM Jeong Y Lee S Im H Tae HS Kim BG. Identification of Beta-Lapachone Analogs as Novel MALT1 Inhibitors to Treat an Aggressive Subtype of Diffuse Large B-Cell Lymphoma. J Med Chem (2015) 58(21):8491–502. doi: 10.1021/acs.jmedchem.5b01415 26496175

[51] Bonsignore L Passelli K Pelzer C Perroud M Konrad A Thurau M. A Role for MALT1 Activity in Kaposi’s Sarcoma-Associated Herpes Virus Latency and Growth of Primary Effusion Lymphoma. Leukemia (2017) 31(3):614–24. doi: 10.1038/leu.2016.239 PMC5339436 27538487

[52] Mc Guire C Elton L Wieghofer P Staal J Voet S Demeyer A. Pharmacological Inhibition of MALT1 Protease Activity Protects Mice in a Mouse Model of Multiple Sclerosis. J Neuroinflamm (2014) 11:124. doi: 10.1186/1742-2094-11-124 PMC4112826 25043939

[53] Van Nuffel E Schmitt A Afonina IS Schulze-Osthoff K Beyaert R Hailfinger S. CARD14-Mediated Activation of Paracaspase MALT1 in Keratinocytes: Implications for Psoriasis. J Invest Dermatol (2017) 137(3):569–75. doi: 10.1016/j.jid.2016.09.031 27939769

[54] Quancard J Klein T Fung SY Renatus M Hughes N Israel L. An Allosteric MALT1 Inhibitor Is a Molecular Corrector Rescuing Function in an Immunodeficient Patient. Nat Chem Biol (2019) 15(3):304–13. doi: 10.1038/s41589-018-0222-1 30692685

[55] Dobin A Davis CA Schlesinger F Drenkow J Zaleski C Jha S. STAR: Ultrafast Universal RNA-Seq Aligner. Bioinformatics (2013) 29(1):15–21. doi: 10.1093/bioinformatics/bts635 23104886 PMC3530905

[56] Trapnell C Roberts A Goff L Pertea G Kim D Kelley DR. Differential Gene and Transcript Expression Analysis of RNA-Seq Experiments With TopHat and Cufflinks. Nat Protoc (2012) 7(3):562–78. doi: 10.1038/nprot.2012.016 PMC3334321 22383036

[57] Kulpa DA Talla A Brehm JH Ribeiro SP Yuan S Bebin-Blackwell AG. Differentiation Into an Effector Memory Phenotype Potentiates HIV-1 Latency Reversal in CD4(+) T Cells. J Virol (2019) 93(24):e00969–19. doi: 10.1128/JVI.00969-19 PMC6880164 31578289

