# Supplementary material for: MALT1-Dependent Cleavage of HOIL1 Modulates Canonical NF-κB Signaling and Inflammatory Responsiveness
Source: Front Immunol. 2021 Oct 14;12:749794. doi: 10.3389/fimmu.2021.749794 (PMC8552041; doi:10.3389/fimmu.2021.749794)
Supplement: Supplementary file 1 [file DataSheet_1.docx]

Supplementary Material

# Supplementary Materials

The supplementary materials include 4 figures and 1 table below.

# Supplementary Figures


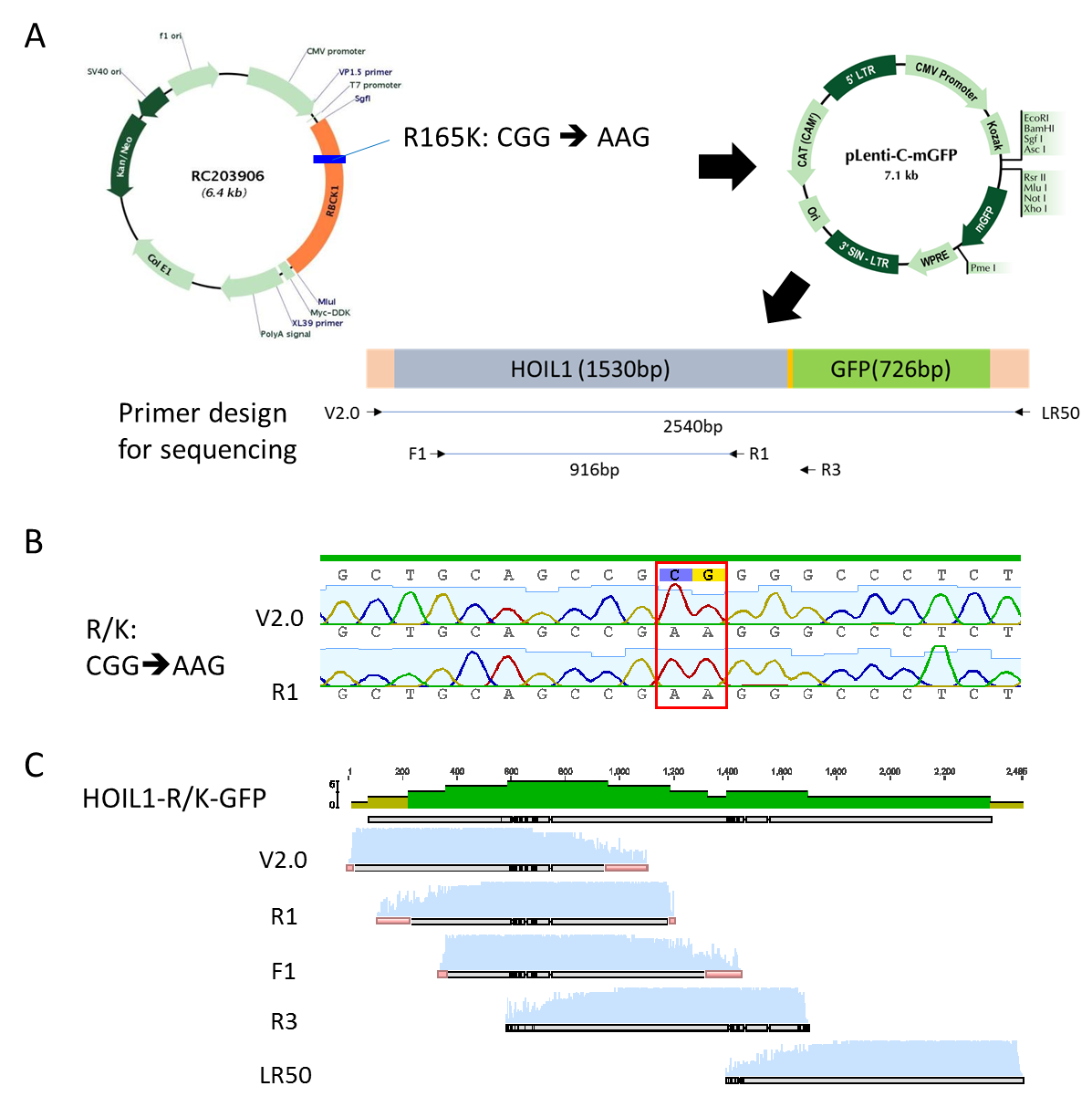


**Supplementary Figure 1.** **Creating a non-cleavable HOIL1 Lenti vector**. (A) Plasmid map showing major features in the entry vector. The mutagenesis R165 site is highlighted in blue (CGG to AAG). The HOIL1 gene was then cloned into an empty Lenti vector with a C-terminal GFP tag. The bottom is a schematic illustrating where the Sanger sequencing primers localize to on the gene. (B) Sanger confirmation of the mutated region (marked by the red box). (C) Full-length sequencing of the construct using 2 forward (V2.0 and F1) and 3 reverse (R1, R3 and LR50) primers. All the primer sequences are listed in the Supplementary Table 1.


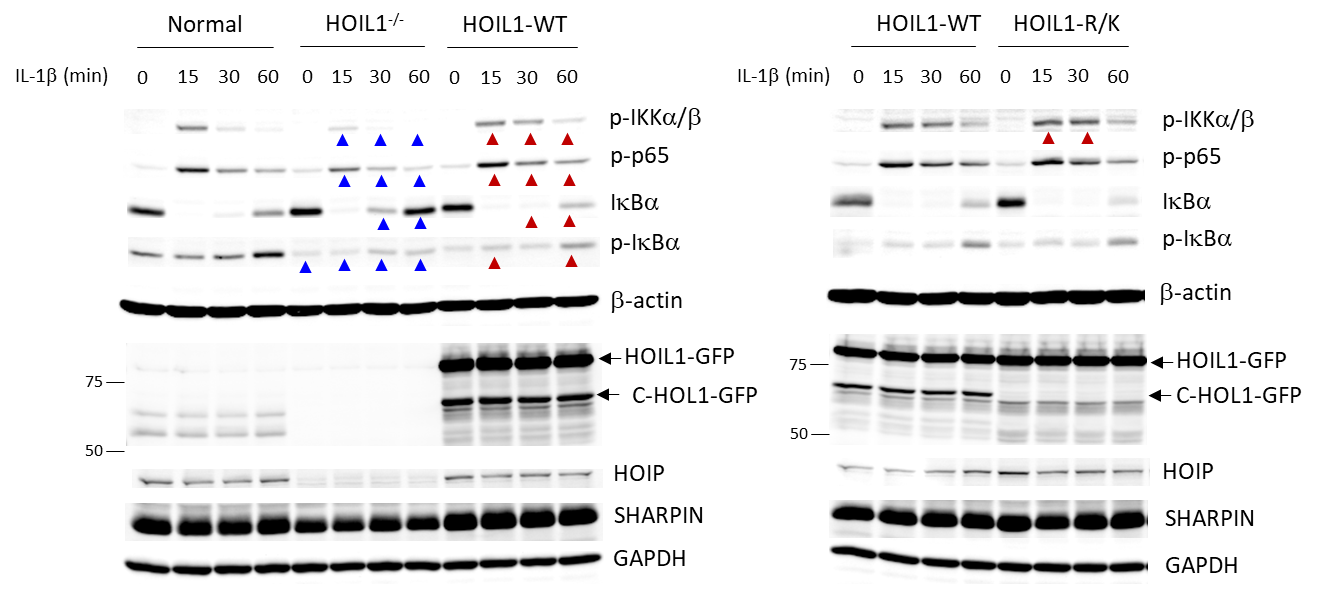


**Supplementary Figure 2.** **Non-cleavable HOIL1 leads to increased canonical NF-κB activation.** The NF-κB activation over time (0-60 min) was probed by the phosphorylation of IKKα/β (p-IKKα/β), p65 (p-p65) and IκBα (p-IκBα) as well as the degradation of IκBα in healthy normal, HOIL1^–/–^, HOIL1^–/–^ overexpressing HOIL1-WT and HOIL1^–/–^ overexpressing HOIL1-R/K fibroblasts. NF-κB was activated by IL-1β in the normal fibroblasts but not in the HOIL1^–/–^ cells; and when the HOIL1^–/–^ cells were reconstituted with HOIL1-WT, the activation of NF-κB was restored (left panel). Reconstituted HOIL1-WT and HOIL1-R/K restored the endogenous HOIP and SHARPIN levels in the HOIL1^–/–^ cells. HOIL1 cleavage (C-HOIL1-GFP) was clearly seen in cells overexpressing HOIL1-WT but not those overexpressing HOIL1-R/K (right panel). β-actin and GAPDH were used as the internal control.


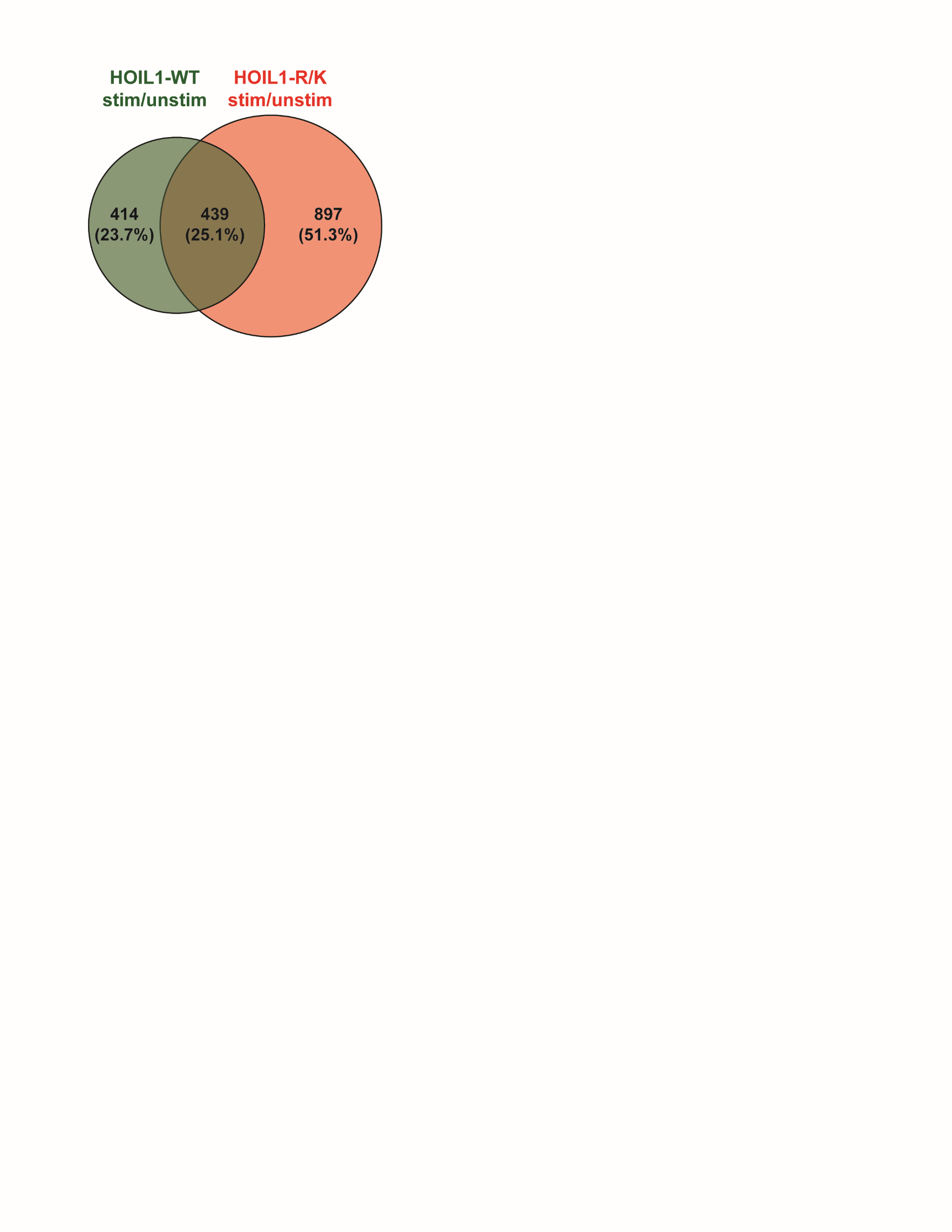


**Supplementary Figure 3.** **Significantly downregulated genes between HOIL1-WT and HOIL1-R/K**. Genes that showed ≤ 1 fold decrease that were significant (adjusted p-val ≤ 0.05) were determined for HOIL1-WT stim/unstim and HOIL1-R/K stim/unstim and compared using Venny.


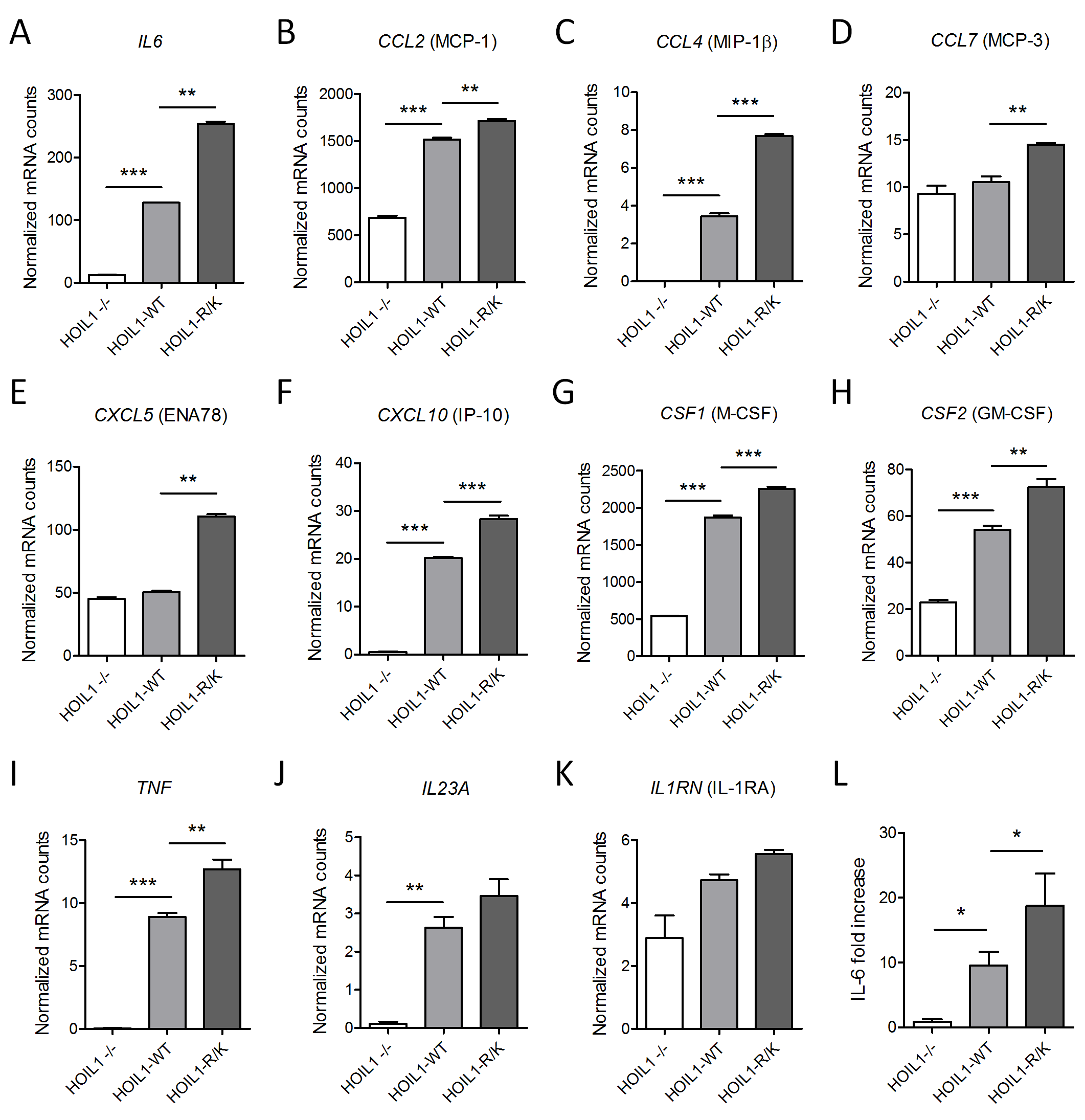


**Supplementary Figure 4.** **Impact of non-cleavable HOIL1 on transcript abundance of proinflammatory cytokines and chemokines**. (A-K) Transcript abundance of *IL6* (A), *CCL2* (MCP-1) (B), *CCL4* (MIP-1β) (C), *CCL7* (MCP-3) (D), *CXCL5* (ENA78) (E), *CXCL10* (IP-10) (F), *CSF1* (M-CSF) (G), *CSF2* (GM-CSF) (H), *TNF* (I), *IL23A* (IL-23) (J) and *IL1RN* (IL-1RA) (K) from RNA-Seq (*N =* 3). (L) IL-6 secretion measured by ELISA (*N* = 3). *: P < 0.05; **: p < 0.01; ***: p < 0.001.

# Supplementary Table

Supplementary Table 1. Primers used for Sanger sequencing

| **Primers** | **Direction** | **Sequence (5’ to 3’)** |
| --- | --- | --- |
| V2.0 | Forward | AGAGCTCGTTTAGTGAA |
| LR50 | Reverse | CAGAGGTTGATTATCGATAAG |
| F1 | Forward | TGACAGTGGCGTCTCTCAAG |
| R1 | Reverse | TCCTCAAAGAAGCACCATCC |
| R3 | Reverse | CTGAACTTGTGGCCGTGC |

Note: The primer location is labeled in the Supplementary Figure 2.
